# [Retracted] Characterization of side population cells isolated from the colon cancer cell line SW480

**DOI:** 10.3892/ijo.2025.5835

**Published:** 2025-12-10

**Authors:** Binghong Xiong, Li Ma, Xiang Hu, Caiquan Zhang, Yong Cheng

Int J Oncol 45: 1175-1183, 2014; DOI: 10.3892/ijo.2014.2498

Following the publication of the above paper, it was drawn to the Editor's attention by a concerned reader that certain of the tumor images shown in Fig. 4A on p. 1180 were unexpectedly similar, albeit the images appeared to have been horizontally stretched where they reappeared in the figure, under situations where different experiments were intended to have been shown.

Owing to the anomalies identified in this figure, the Editor of *International Journal of Oncology* has decided that this paper should be retracted from the Journal. The authors were asked for an explanation to account for these concerns, but the Editorial Office did not receive a reply. The Editor apologizes to the readership for any inconvenience caused.

